# The Role of Prostaglandins as Major Inflammatory Mediators in Colorectal Cancer

**DOI:** 10.3390/ijms262412191

**Published:** 2025-12-18

**Authors:** Mario Macia Guardado, Valentina Lutz, Markus Hengstschläger, Helmut Dolznig

**Affiliations:** 1 Center of Pathobiochemistry and Genetics, Institute of Medical Genetics, Medical University of Vienna, Waehringer Strasse 10, 1090 Vienna, Austria; mario.maciaguardado@meduniwien.ac.at (M.M.G.);; 2 Comprehensive Cancer Center (CCC) Vienna, Medical University of Vienna, 1090 Vienna, Austria

**Keywords:** colorectal cancer, inflammation, prostaglandins, microenvironment

## Abstract

Colorectal cancer (CRC) is one of the leading causes of cancer-related morbidity and mortality, with inflammation playing a pivotal role in its pathogenesis. Chronic inflammation in the intestine significantly increases the risk of CRC development. Main compounds participating in the inflammatory process are prostaglandins; bioactive lipids derived from arachidonic acid metabolism via the cyclooxygenase (COX) pathway. While it is well known that prostaglandin E_2_ (PGE_2_) promotes CRC tumorigenesis, other prostaglandins, such as PGD_2_, PGF_2α_, and prostacyclin (PGI_2_), remain relatively underexplored. These prostaglandins may exert distinct or opposing effects on CRC development, but the current understanding of their functions is limited. Additionally, the impact of prostaglandins on immune regulation and the tumor microenvironment, is far from being fully understood. Addressing these knowledge gaps is crucial for identifying novel therapeutic targets and optimizing chemoprevention strategies. Non-steroidal anti-inflammatory drugs (NSAIDs) have been shown to reduce the risk of CRC, largely by inhibiting prostaglandin producing enzymes. However, their use is limited due to their gastrointestinal and cardiovascular side effects. Therefore, understanding the intricate role of inflammation and prostaglandin signaling in CRC is critical to develop safer and more effective chemopreventive approaches. This review summarizes the current knowledge of prostaglandins, linking inflammation and CRC. It further addresses the potential of targeting prostaglandin pathways for chemoprevention. Furthermore, we discuss emerging pharmacological targets that modulate prostaglandin production, signaling or degradation, offering promise for preventing CRC development.

## 1. Introduction

Colorectal cancer (CRC) is one of the most common types of cancer worldwide. According to GLOBOCAN 2022, CRC is the third most commonly diagnosed cancer worldwide, making up 9.6% of all new cases, and the second leading cause of cancer-related deaths, responsible for around 9.3% of all cancer fatalities [1]. Even though the increased use of screening techniques has decreased the incidence and mortality of CRC, there is still a high number of patients (approximately between 25 and 50%) that will develop metastases after an early-stage diagnosis. Approximately 25% of the CRC patients are going to be diagnosed at an already advanced stage of the tumor development [2]. Thus, there is urgent need for novel treatment options to increase survival in CRC [3]. A plethora of different factors are associated with the development of CRC, such as age, a Western diet based on red and processed meat and low in fiber consumption [4], obesity [5], lack of exercise (sedentary lifestyle) [6], alcohol consumption [7], and smoking [8]. Moreover, inflammation and genetic factors also contribute to CRC development, with approximately 10–20% of patients having a positive family medical history [9]. These factors have been all linked to a higher incidence of CRC and play a major role in its development [10,11].

## 2. Inflammation

It is clinically well known that inflammatory bowel disease (IBD), such as ulcerative colitis and Crohn’s disease are linked to the development of CRC. This discovery set the base to investigate how inflammation is able to promote tumor development [12]. Thus, in order to understand the role of prostaglandins as major inflammatory mediators in tumorigenesis we need to first understand the basic principles of inflammation and the involvement of prostaglandins in the inflammatory process. However, we do not intend to give a precise general review on inflammation, as this was excellently done elsewhere [13,14,15]. Inflammation is the body’s response to tissue damage caused by infection, injury, or ischemia [16]. It is closely linked to wound healing, serving as its initial phase. This response triggers changes at cellular and systemic levels to protect and repair damaged tissue [17]. While acute inflammation is beneficial, chronic inflammation can contribute to diseases such as diabetes, Alzheimer, and cancer [18]

### 2.1. Acute Inflammation

**Figure 1:**
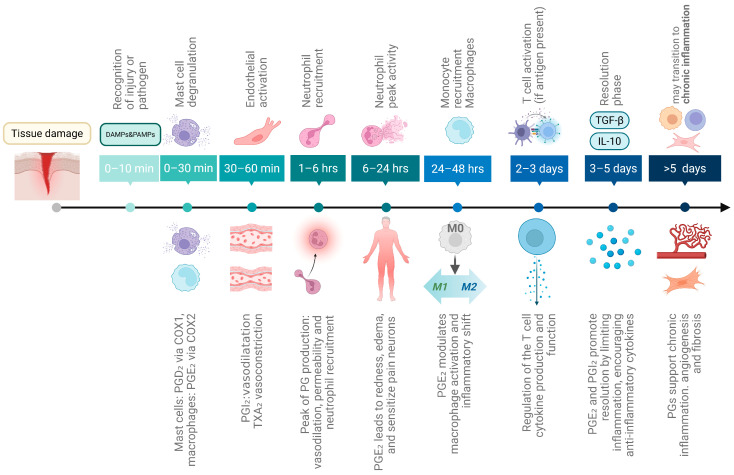
**Timeline of acute inflammation and its role in CRC development.** The scheme displays the sequence of cellular and molecular events during acute inflammation, beginning with tissue damage or infection. If unresolved after six weeks, inflammation progresses into a chronic state, marked by persistent tissue damage, fibrosis, and repeated cycles of damage and repair that promote cell proliferation and may ultimately lead to CRC development. Above, the cellular and molecular events of acute inflammation are shown, while below the modulatory contribution of prostaglandins at each stage is depicted. Created in Biorender. Helmut Dolznig (2025) https://BioRender.com/se51lfr.

Acute inflammation is a short-term, self-limiting response to injury or infection (Figure 1, top), that initiates tissue repair [19]. Resident immune cells (e.g., mast cells, macrophages, dendritic cells) identify pathogens or damage using pattern recognition receptors (PRRs). PRRs (like TLR4) [20,21] detect pathogen-associated molecular patterns (PAMPs) or, in cases of non-infectious injury, damage-associated molecular patterns (DAMPs) [22,23]. Early responses include mast cell degranulation, release of histamine, heparin, TNF-α and proteases, followed by vasodilation, and increased vascular permeability, which quickly enhance blood flow inducing endothelial activation and facilitating immune cell recruitment. Nitric oxide (NO), released by endothelial cells and phagocytes, further amplifies inflammation and helps control pathogens, though excessive NO can contribute to tissue damage [24,25]. Further, pro-inflammatory cytokines are produced by mast cells, endothelial cells and macrophages at injury sites. These cytokines play key roles in initiating, sustaining and resolving inflammation during tissue repair, being secreted also by fibroblasts and epithelial cells [17,26,27]. Cytokines trigger acute-phase responses, including increased production of proteins like C-reactive protein and fibrinogen in the liver, and reduced levels of zinc, iron, and albumin [28]. Excessive cytokine release can provoke a systemic inflammatory “cytokine storm” [29].

Neutrophils are the first leukocytes to arrive at the site of inflammation (within 1–6 h). After reaching the inflammation site [30] they eliminate pathogens and damaged cells via degranulation ROS and antimicrobial enzyme release, NET formation, and phagocytosis [31]. ROS not only directly kill pathogens but also amplify PAMP/DAMP signaling, inflammasome activation, oxidative stress meditated cell death and immune cell recruitment. Neutrophil peak activity is reached between 6 and 24 h.

Monocytes are recruited to inflammation sites via chemokine action, 24 to 48 h after neutrophils. They differentiate into macrophages or dendritic cells, clearing pathogens, dead cells and debris. One main function is to clean up apoptotic neutrophils [32] by efferocytosis. After engulfment, macrophages and dendritic cells can present antigens to T cells, initiating adaptive immunity. Macrophages and monocytes secrete pro-inflammatory cytokines, ROS and NO, which amplify the immune response. After 3–5 days, inflammation shifts toward the resolution phase [33], marked by anti-inflammatory molecules like IL-10, TGF-β, Annexin A1, and specialized pro-resolving mediators (SPMs) like resolvins, protectins, lipoxins, and maresins [34]. Fibroblasts and endothelial cells also contribute to tissue repair.

There are many other important aspects, cells or mechanism of acute inflammation, which are not primarily associated with the topic here and thus are not addressed due to space restrictions.

### 2.2. The Timing of Prostaglandin Involvement in Acute Inflammation

It remains to clarify, how prostaglandins (PGs) are involved in the inflammatory process (Figure 1 bottom). Detailed information on PG synthesis and function is presented below in a separate chapter. Under normal circumstances, PG production is generally very low, but is substantially increased during acute inflammation even prior to leukocyte recruitment and immune infiltration [35]. Mast cells are the first cells to release PGs (especially PGD_2_) upon degranulation within minutes after injury. This initial PG source is thought to be mediated by cyclooxygenase 1 (COX-1) [36]. Then, resident macrophages activated rapidly by DAMPs/PAMPs produce mainly PGE_2_ but also other eicosanoids via inducible COX-2. Endothelial cells produce mainly PGI_2_ (prostacyclin) to promote vasodilation and to counteract platelet aggregation and leukocyte adhesion [37]. PGE_2_ contributes to vasodilation and increased microvascular permeability, leading to redness and edema [38]. Simultaneously, pain is induced by PG action on peripheral sensory neurons and central sites of the brain [39,40]. PGE_2_ also promotes dendritic cell migration [41]. In contrast, platelets produce thromboxane A_2_ (TXA_2_) opposing PGI_2_ function and leading to vasoconstriction, platelet aggregation and smooth muscle contraction [42], thereby balancing clotting and flow. PG production is peaking at about 1 to 3 h. Cyclooxygenase 2 (COX-2) is the key enzyme stimulating PG production in this process, as its inhibition reduces PG levels and suppresses inflammation at early stages [43].

### 2.3. Chronic Inflammation

If acute inflammation fails to resolve [16] and persists beyond several days to >3 weeks [44], it transitions to chronic inflammation. Persistent inflammation leads to tissue damage and fibrosis [45]. Chronic inflammation can lead to sustained collateral cell damage such as apoptosis, necrosis and to the accumulation of mutations [46,47]. In an attempt of the body to counteract the injury, continuous tissue repair and cell proliferation is induced. Additionally, it is characterized by the permanent production of pro-inflammatory cytokines and lipids, such as PGs, an insufficient production of SPMs and recruitment of inflammatory cells while inhibiting regulatory T cells (Treg) [48,49]. Macrophages and neutrophils continuously release ROS and cytokines and degrade the extracellular matrix (ECM) and disrupt tissue integrity [50,51].

Chronic inflammation also leads to a continuously activated wound healing process, largely mediated by fibroblasts. After injury, immune cells secrete cytokines like TGF-β1, TNF-α, IL-6, and PDGF [52,53], which recruit and activate fibroblasts inducing differentiation into myofibroblasts, the principal ECM-producing cells [54,55]. They produce collagen and fibronectin, creating the stiff ECM typical of fibrotic lesions [56]. Excessive ECM deposition, called fibrosis, distorts the tissue and can lead to organ failure [52], contributing to nearly 45% of deaths in developed countries [57]. Macrophages support fibrosis by expressing profibrotic genes and sustaining fibroblast activation [58,59]. Chronic inflammation also promotes a prothrombotic state via neutrophil-platelet interactions, a common feature in IBD [60] and ulcerative colitis [61].

### 2.4. Inflammation and Its Impact on Cancer Development and Progression

Inflammation sometimes promotes the transformation of early-stage neoplasia into fully developed cancers and is considered a hallmark of cancer. Chronic inflammation drives increased cell proliferation and DNA damage exposure, leading to tumor development [14] through mutations (e.g., in TP53) and genomic instability. Especially, persistent amounts of ROS are involved in this process [62]. In recent years, the focus was shifted to a holistic understanding of cancer ecology, underscoring the complex interplay between inflammation and tumorigenesis, including epigenetically adaptable cancer cells and the tumor microenvironment (TME), such as fibroblasts or immune cells [63]. The continuous presence of inflammatory signals affects the TME itself, which in turn facilitates tumor development [64,65,66]. Chronic inflammation is one of the major biological characteristics of non-benign tumors [67,68]. Inflammation contributes to the interaction of the cells that constitute the TME thereby influencing tumor development and the plasticity of the cancer cells [63]. Some examples of chronic inflammation that are known to be a risk factor of developing cancer are, e.g., chronic gastritis after infection with *helicobacter pylori* leading to the development of gastric cancer [69], liver cancer after infection with the hepatitis B and C virus [70] and induction of CRC in patients that suffer from IBD [71], which is discussed in more detail below.

Interestingly, acute inflammation can counteract cancer progression, as it is generally considered that it triggers a robust antitumor immune response, which can lead to the regression or complete elimination of tumors [72]. However, there is also growing evidence that acute inflammation can also contribute to cancer [73].

#### Role of Inflammation in CRC

Basically, there are three forms of inflammation in CRC classified based on the moment at which inflammation influences CRC pathogenesis: (1) chronic inflammation that precedes tumorigenesis; (2) tumor-elicited inflammation; and (3) therapy-induced inflammation. The three forms share the common feature of promoting tumor growth by activating innate immune cells and creating an immunosuppressive TME; however, they might play different roles depending on the tumor progression stage [74]. They also apply for other cancer types; CRC is used to exemplify this classification.

As pre-tumoral chronic inflammation  is concerned, environmental factors, such as diet or lifestyle, infections or long standing IBD can trigger chronic inflammation that increases the possibility of developing CRC [75]. IBD is clearly linked to the development of CRC. Crohn’s disease and ulcerative colitis share roughly 30% of IBD-related genetic loci showing that they act, at least in part, via similar pathways. These pathways include loss of barrier function, epithelial restitution, microbial defense, innate immune regulation, ROS generation, among others associated with the homeostasis of the intestine [76]. The origin of IBD is not clearly understood but thought to be initiated from an abnormal and continuous inflammatory response to commensal microbiota. Family history is an important risk factor associated with the progression of IBD [76]. Individuals suffering from these diseases, have an estimated 2–3 fold increased risk of CRC [77]. Thus, during the last few decades, it has been established that IBD is an independent risk factor for CRC. As the disease persists over time and chronic inflammation is causing cumulative damage in the colon, the likelihood of developing CRC increases significantly [78]. IBD is characterized for showing multiple mutations in genes associated with immune activation and response [76]. STAT3, whose gene encodes a locus involved in IBD, is activated in epithelial cells of IBD patients [79]. IL-22, a cytokine involved in STAT3 activation, displays a protective effect on epithelial cells in different colitis models. In Crohn’s disease there is a lower frequency of IL-22-secreting innate lymphoid cells in the lamina propria [80,81]. Primary CRC is also often associated with inflammatory cell infiltrates, associated by the increased expression of inflammatory factors [82].

For tumor elicited inflammation it is noteworthy to mention that while inflammation can strongly drive tumor initiation as mentioned above, most cancers arise without being preceded by noticeable inflammation [83]. In tumor-elicited inflammation, tumor progression triggers an inflammatory response that is often pro-tumorigenic, driven by hypoxia-induced cell death or the disruption of the epithelial barrier, which allows microbial organisms or their products to infiltrate [84].

Third, therapy-induced inflammation is caused as a response to different anti-cancer therapies, including chemo- and radiotherapy. They can promote tumor-supportive processes by altering the TME, triggering cellular “wound-healing” responses [85]. While inflammation resulting from therapy is often an unintended consequence rather than a primary goal, it plays a significant role in shaping therapeutic outcomes and the likelihood of relapses.

## 3. Prostaglandins

PGs play a key role in CRC development among the factors involved in the inflammatory response and promote the classic signs of inflammation: *rubor*, *calor*, *tumor* and *dolor* [35]. As already outline above, PGs are promptly induced: their levels rise rapidly in acute inflammation, even before immune cell infiltration [86].

### 3.1. General Overview and Classification

**Figure 2:**
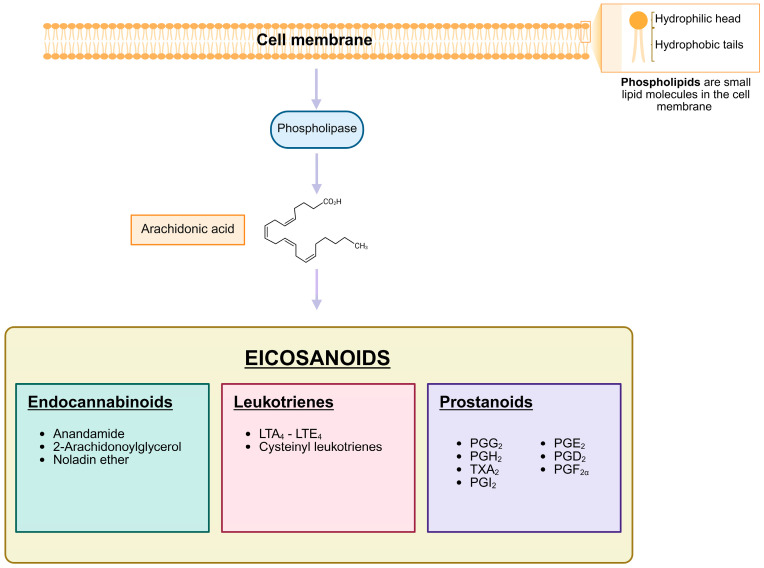
**Biosynthesis and Classification of Eicosanoids**. The scheme depicts the biosynthetic pathway of eicosanoids, which are lipid-derived signaling molecules. Membrane phospholipids are enzymatically converted into arachidonic acid, a key precursor for the synthesis of eicosanoids. These are categorized into three major classes: endocannabinoids, including anandamide, 2-arachidonoylglycerol, and noladin ether; leukotrienes, such as LTA_4_, LTB_4_, LTC_4_, LTD_4_, and LTE_4_, which include cysteinyl-leukotrienes; and prostanoids, encompassing prostaglandins, prostacyclins, and thromboxanes. Each class plays a distinct role in physiological and pathological processes, such as inflammation, immune regulation, and vascular homeostasis. Created in Biorender. Helmut Dolznig (2025) https://BioRender.com/qjgakvr.

PGs are a subgroup of the eicosanoids, along with the leukotrienes, and classified as prostanoids together with the thromboxanes and prostacyclin [87] (Figure 2). Like all eicosanoids, PGs are a group of lipid compounds with 20 carbon atoms that are derived from arachidonic acid (AA), a polyunsaturated fatty acid [87,88]. They were discovered in the 1930s by Swedish physiologist Ulf von Euler in seminal fluid. He assumed that they originated in the prostate gland leading to the name of “prostaglandins” [89]. PGs have hormone-like properties that act as autocrine and paracrine messengers and are synthesized by a diverse range of cells in the whole organism, except for red blood cells. They have a short half-life, typically in the range of seconds to a few minutes after they are produced because of their rapid degradation and inactivation by 15-hydroxyprostaglandin dehydrogenase (15-PGDH). This fast degradation is crucial for their confined function, PGs act locally on nearby cells rather than traveling through the bloodstream [90,91]. PG function is organ- and tissue-specific. PGs exert their biological function by binding to G protein-coupled receptors (GPCRs) named prostanoid receptors [92] and initiating downstream signaling.

### 3.2. PGs Synthesis: Pathway and Mechanisms

**Figure 3:**
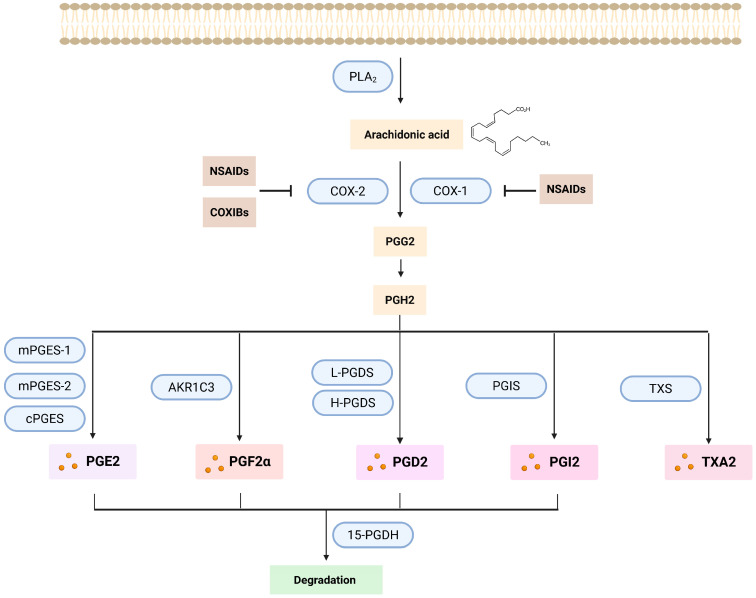
**Prostaglandin synthesis pathway.** PGs originate from the AA released from the phospholipids of the cell membrane by the action mainly of PLA2. AA is then transformed into PGG2 by the action of the COX enzymes (COX-1 and COX-2). These two enzymes can be inhibited by the action of the NSAIDs (in the case of both of them) or COXIBs (only COX-2). By deperoxidation PGG2 is transformed into PGH2, which is then converted by different specific synthases to the main active prostanoids: PGD2, PGE2, PGF2α, prostacyclin PGI2, and thromboxane TXA2. Subsequently, all prostanoids, except TXA2, will be oxidized by the enzyme 15-PGDH, thereby inactivated and eliminated. Created with Created in Biorender. Helmut Dolznig (2025) https://BioRender.com/als0wtb.

As mentioned above, PGs are synthesized from AA, which is derived from the phospholipid bilayer of the cell membrane. Proinflammatory factors are triggering the initiation of the PG synthesis pathway (Figure 3) by activating the phospholipases A2 (PLA2) and C (PLC), resulting in the release of AA from the cell membrane [93]. The release of AA is predominantly regulated by the expression level and/or activity of PLA2. Once activated, PLA2 can translocate to the membrane where it hydrolyzes the ester bond at the sn-2 position of the phospholipids releasing free fatty acids such as AA [94]. AA disseminates through the cytosol until metabolized by the prostaglandin endoperoxide synthases (PTGS), also known as cyclooxygenases (COX) [87,95]. The COX enzymes in humans are present in 2 isoforms: COX-1 is constitutively expressed in most cell types and encoded by the PTGS1 gene [96]. COX-1 plays an important role in normal physiology, such as gastrointestinal protection and platelet aggregation [93,96]. COX-2 is encoded by PTGS2 [96] and it is linked to proinflammatory stimuli. It is expressed at low levels in normal tissue [97]. Both are membrane proteins constituted of four protein domains [87,95] and localized to the luminal surface of the ER and the membranes of the nuclear envelope [98]. The maturation of the COX enzymes takes place in the ER [96]. They are integrated into the ER cell membrane, creating effective homodimers, that will help AA to enter their active site for catalysis through an access channel. The biosynthesis of all the prostanoids is catalyzed by the COX enzymes in a rate limiting step reaction initiated by the oxygenation of the carbons at positions 11 and 15 of the AA which leads to the production of prostaglandin G2 (PGG_2_), which is an unstable and short-lived molecule [99]. Then, a deperoxidation takes place by the peroxidase activity of COX, leading to the formation of the direct precursor of all the prostanoids, prostaglandin H2 (PGH_2_). This process occurs mainly in the ER [87,98]. This intermediate will then be converted, by specific synthases located in the ER membrane or in the cytosol, into the main active prostanoids: PGE_2_, PGF_2α_, PGD_2_, prostacyclin (PGI_2_) and thromboxane A_2_ (TXA_2_) (Table 1) [99].

Three distinct PGE_2_ specific synthases have been identified: two microsomal isoforms, microsomal prostaglandin E synthase-1 (mPGES-1) and mPGES-2, and a cytoplasmic variant, cPGES [100]. The term microsomal refers to the fact that it can be found in the microsomal fraction after subcellular fractionation [101,102]. Thus, the microsomal forms of PGE_2_ synthases are predominantly located in the ER. The PGF_2α_ synthesis pathway is more complex. There are three distinct biosynthetic pathways involved in the production of PGF_2α_. The main synthesis pathway is via the 9,11-endoperoxide reduction in PGH2 by the enzyme aldo-keto-reductase 1 C3 (AKR1C3), but PGF_2α_ can as well be produced by the 9-ketoreduction of PGE_2_ and the 11-ketoreduction of PGD_2_ [103]. The production of PGD_2_ is mainly carried out by two synthases: lipocalin-like PGD synthase (L-PGDS) and hematopoietic PGD synthase (H-PGDS), which convert PGH2 into PGD_2_. [91,103]. PGD_2_, PGE_2_ and PGF_2α_ can be found in most human organs; this is why they are occasionally designated as the “housekeeping” PGs. On the other hand, PGI_2_ and TXA2, which are synthesized primarily by endothelial cells and thrombocytes, respectively, are viewed as more specialized [104].

PGs synthesized de novo cannot efficiently cross biological membranes by passive diffusion. The PG transport can be actively facilitated by specific transporters, such as the prostaglandin transporter (PGT, SLCO2A1), known for its high affinity for PGs [105,106]. This transporter has been shown to regulate the distribution of PGs, particularly PGE_2_. It is a high-affinity, electrogenic anion exchanger that facilitates the concentrative uptake of PGs in exchange for intracellular anions, mostly lactate [106]. As already pointed out above, due to their rapid breakdown, eicosanoids primarily exert their effects locally, near the site where they are produced [107].

**Figure 4:**
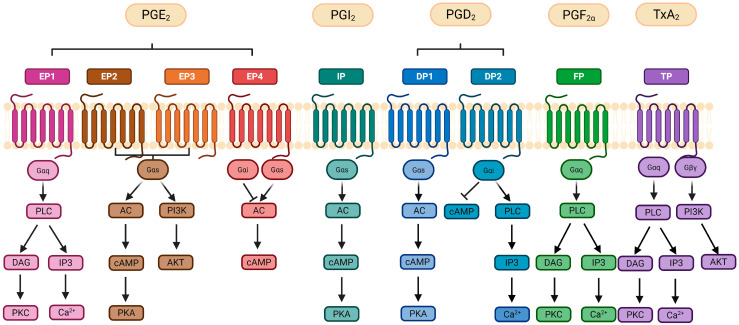
**Signaling pathways initiated by prostanoid release.** Activation of G-protein-coupled receptors (GPCRs) involves the exchange of guanosine triphosphate (GTP) for guanosine diphosphate (GDP), leading to dissociation of the alpha (α) subunit from the beta and gamma (β/γ) subunits and initiation of downstream signaling pathways. Prostanoid GPCRs activate distinct pathways: EP1, FP, and TP receptors use Gαq to stimulate phospholipase C (PLC), generating inositol triphosphate (IP3) and diacylglycerol (DAG), which elevate intracellular Ca^2+^ and activate protein kinase C (PKC). EP2/EP4, IP, and DP1 receptors couple to Gαs, activating adenylate cyclase (AC), increasing cyclic AMP (cAMP), and stimulating protein kinase A (PKA). Dysregulated EP2/EP4 signaling can also activate PI3K/AKT and MAPK/ERK pathways, promoting proliferation and survival. EP3 signaling depends on its splice variant, with the most common variant reducing cAMP via Gαi. Similarly, DP2 links to Gαi, which inhibits cAMP or activates PLC, while TP also recruits Gβγ for PI3K/AKT signaling. Abbreviations: AC (adenylate cyclase); AKT (protein kinase B); DAG (diacylglycerol); ERK (extracellular signal-regulated kinase); IP3 (inositol triphosphate); MAPK (mitogen-activated protein kinase); PI3K (phosphoinositide 3-kinase); PKC (protein kinase C); PLC (phospholipase C); Ca^2+^ (calcium ion); GTP (guanosine triphosphate). Created in Biorender. Helmut Dolznig (2025) https://BioRender.com/wnsjxl3.

Each prostanoid has its own associated receptor (Figure 4) constituting a total of nine receptors: the two PG D receptors (DP1 and DP2), four subtypes of the PG E receptor (EP1, EP2, EP3 and EP4), the PG I receptor (IP), the PG F receptor (FP) and the thromboxane receptor (TP). On top of that, the EP3, FP and TP receptors exist in different splice variants that differ in their C-terminus [108]. Each receptor shows higher affinity for one type of PG. Overall, the coupling of the prostanoids to the specific receptors promotes proliferation and invasion of cancer cells; it inhibits apoptosis and promotes survival [91,109]. Each receptor shows a varying cellular expression profile in tissues, being differentially distributed in the organism. For instance, in mice, EP2 is the least abundant receptor, whereas EP3 and EP4 are the most widely distributed. In contrast, EP1 mRNA expression is largely confined to specific organs such as kidney, stomach and lung [110,111]. Activation of EP1 (405 amino acids [aa]) receptors mainly mobilize calcium (Ca^2+^). In addition, the interaction between PGE_2_ and the EP1 receptor activates multiple signaling pathways, including EGFR, PI3K, MAPK and NF-κB, which contribute to cancer cell proliferation, invasion and migration [91]. Activated EP2 and EP4 increase the intracellular cyclic adenosine monophosphate (cAMP) levels. On the other hand, active EP3 reduces cAMP [112]. Interaction of PGE_2_ with the EP2 (362 aa) receptor, which is upregulated by COX-2-driven inflammation, can lead to the expression of proinflammatory factors such as IL-1β. EP3 exists in several splicing isoforms which differ mainly in their C-terminal region, including EP3α (366 aa), EP3β (362 aa), and EP3γ (365 aa) [111]. This receptor inhibits adenylyl cyclase by coupling with Gi, leading to a decrease in cAMP levels. These differences in the C-terminal region allow each isoform to interact with different G-proteins and trigger distinct signaling pathways inside cells. As a result, the various EP3 isoforms can produce different effects depending on the tissue and context, some may increase or decrease cAMP, or affect other signaling molecules. This diversity helps explain why PGE_2_ can have complex and sometimes opposite effects in inflammation and disease [113]. Conversely, activation of the EP4 (513 aa) receptor plays a role in several signaling pathways involved in cancer development and progression, influencing processes such as proliferation, apoptosis, migration, and metastasis. Specifically, EP4 activation can trigger the PKA/PI3K/AKT pathway, which promotes cell proliferation [91,114]. EP4 receptors are the most abundantly expressed PGE_2_ receptors [115].

After joining their respective receptors and initiating downstream signaling, PGs are degraded by the enzyme 15-PGDH. First, they are transported into the cell via the PGT, mentioned above, which is located on the plasma membrane, and, thereafter, 15-PGDH, which is present in the cytosol of the cell, oxidizes and inactivates them [87]. SLCO2A1 mediated uptake of PGE_2_ is the rate-limiting step in its degradation, and inactivation by 15-PGDH occurs quickly after entry [116].

### 3.3. The Role of PGs in the Homeostasis of the Organism and Non-Malignant Diseases

PGs are involved in a broad range of biological roles: from maintaining homeostasis to playing key roles in inflammation, immune modulation, blood pressure regulation, fever and pain [87,88,92].

PGE_2_ is involved in the regulation of immune response, gastrointestinal integrity, modulation of blood pressure, fertility, tissue regeneration, inhibition of fibrosis and bone formation [35,117].

PGF_2α_ is expressed in various tissues and is mainly involved in the female reproductive system [92,118], regulating uterine contractions during menstruation, childbirth, and ovulation. It also affects kidney function [119] and its receptor agonist, latanoprost, reduces intraocular pressure in glaucoma [35,120]. Also, PGF_2α_ is a well-known inhibitor of adipocyte differentiation [121]. PGF_2α_ normally exerts its action by joining its membrane receptor: the FP receptor (FP, encoded by the gene PTGFR) [122]. FP function in the human colon remains poorly understood [123,124]. PGF_2α_ is synthesized by aldo-keto reductases (AKRs), a family of 15 enzymes [118]. AKR1C3 converts PGD_2_ into PGF_2α_ [125], while AKR1C1 and AKR1C2 interconvert PGE_2_ and PGF_2α_ [126]. In vivo, two stereoisomers of PGF_2α_ exist: one derived from PGH_2_ and PGE_2_, and 11β- PGF_2α_ originating from PGD_2_ [103], which binds FP, EP1 and EP3 receptors with high affinity.

While some studies show that PGD_2_ participates in the resolution of inflammation [127,128] it can also play a role in the development of diseases related to IgE-mediated type 1 acute allergic response [129,130]. It is involved in reproduction [131] and it is implicated in the sleep cycle regulation and pain perception [132,133]. PGD_2_, as explained above, exerts its action by joining DP1 and/or DP2 receptors having a dual role: (1) resolution of inflammation via DP1; (2) contribution to IgE-mediated allergic responses via DP2 activation on Th2 lymphocytes, eosinophils, and basophils [134]. DP2 triggers inflammatory cell recruitment in diseases like asthma [135] and atopic dermatitis [136].

PGI_2_ and TXA2 participate in the regulation of blood flow and clotting, being described as key factors in the cardiovascular system. While PGI_2_ acts as a vasodilator inhibiting platelets aggregation and, therefore, blood clotting, TXA2 plays the opposite role, being synthesized and released by the platelets themselves [137] and supporting blood clotting and vasoconstriction [138]. TXA2 is also a potent bronchoconstrictor during asthma and promotes the proliferation of airway smooth muscle cells [139]. TXA2 influences multiple biological processes through its binding to its specific receptor (TP), and subsequently activating multiple downstream pathways due to a rise in intracellular Ca^2+^ levels [140].

In general, a dynamic balance between PG production and PG degradation is needed in order to maintain the organism homeostasis. Dysregulation of this balance is associated with the development of both benign and malignant diseases as described below in detail.

### 3.4. PG in Pathology: Influencing Cancer

#### Induction of COX-2 in Tumors and COX-2 as a Driver of Tumor Progression

As already outlined briefly above, cancer and chronic inflammation are closely connected. Activation of COX-2 by proinflammatory mediators at the inflammation site [68], plays and important role, as COX-2 is overexpressed in 85–90% of tumors [141], but not necessarily in tumor cells. For example, IL-1β and TNF-α can enhance PGE_2_ production by up to 25-fold in human colorectal fibroblasts [142].

Several studies have shown that NF-kB, the master modulator of gene expression associated with chronic inflammation, regulates COX-2 expression. The NF-κB protein family comprises five members: NF-κB1 (p50), NF-κB2 (p52), RelA (p65), RelB, and c-Rel [143]. Among these, p65 plays a key role in COX-2 regulation in cancer cells [144]. NF-kB translocates to the nucleus after being activated and joins the binding site in the COX-2 promoter region, subsequently stimulating COX-2 expression [145]. In osteosarcoma a positive feedback loop has been described, in this case COX-2 overexpression induces NF-kB expression [146]. Additionally, proinflammatory cytokines such as IL-1β and TNF-α contribute to the activation of the mitogen-activated protein kinase (MAPK) pathway, which subsequently plays a crucial role in the activation of NF-κB [68]. Furthermore, the activation of p38 MAPK by pro-inflammatory stimuli also promotes the upregulation of phospholipases A2, which in turn increases the release of AA [147]. COX-2 promotes the phosphorylation of PI3K, AKT and IKK proteins, subsequently leading to increased cell proliferation, metastasis, drug resistance development and involvement in angiogenesis.

Noteworthy, TGF-β, a major driver of EMT, is atypically expressed in approximately 90% of tumors that also show overexpression of COX-2 [141]. TGF-β was shown to promote the nuclear accumulation of β-catenin [148] and COX-2 is also a transcriptional target of the Wnt/β-catenin signaling pathway. Thus, TGF-β can enhance COX-2 protein levels, in concert with stimulating invasiveness and metastasis in advanced tumor stages, as TGF-β typically stimulates cells to undergo EMT [141].

The impact of oxidative stress on mutations and tumorigenesis is well established, and there is a link to PG signaling. High levels of ROS not only stimulate cancer cell progression but also affect the TME [149]. ROS regulates NF-κB and facilitates the activation of the Wnt pathway. Elevated levels of ROS lead to a rise in pro-inflammatory cytokines, such as IL-1β and TNF-α. Furthermore, ROS activates MAPK/ERK kinase 1, which collectively boosts COX-2 expression [149]. Simultaneously, the peroxidase activity of COX-2 generates ROS, making ROS both an activator and a byproduct of COX-2 activity and creating a feedback loop between ROS and COX-2 [150].

Hypoxia is another factor that stimulates COX-2 in cancer [151]. The activation of the hypoxia inducible factor (HIF) pathway plays a role in the inflammatory response activating COX-2 in a HIF dependent manner. It has been demonstrated that a hypoxic environment upregulates COX-2 in CRC cells [152]. In line with this observation, a functional hypoxia response element has been discovered in the COX-2 promoter sequence [153,154]. Counterintuitively, there is a role of ROS in hypoxia, although oxygen is necessary for the generation of ROS. Hypoxic conditions can alter the cellular supply of NADPH activating NADPH oxidases leading to mitochondrial malfunction. NADPH is essential for maintaining reduced glutathione levels in the presence of chemical oxidants. This disruption of NADPH may result in an overall increase in cellular oxidative stress under hypoxic conditions in specific contexts enhancing ROS production. Overall, more research is needed to determine the specific mechanism that increases ROS production under low oxygen conditions [155,156,157].

COX-2/STAT3 signaling promotes cancer cell proliferation and epithelial–mesenchymal transition (EMT), supporting an immunosuppressive TME. Also, it has been shown that COX-2 activates STAT3 by inducing IL-6 expression in lung cancer [158,159]. Another example is SDF-1α (CXCL12), which by activating its receptor CXCR4 facilitates cancer metastasis and invasion by stimulating COX-2 [160,161]. Additionally, COX-2 undergoes several post-translational modifications that influence its enzymatic activity and its susceptibility to degradation [95]. Overall, induction of COX-2 expression plays a crucial role in cancer progression by enhancing cell proliferation, stimulating angiogenesis, inhibiting apoptosis and increasing metastatic potential [68].

### 3.5. Role of Individual PGs in CRC Pathology

#### 3.5.1. PGE_2_ and EP Receptor Signaling in CRC

The primary effects of COX-2 are largely due to the release of PGE_2_, which is thought to mediate many of COX-2’s tumor-promoting actions [162]. PGE_2_ is well-established to play a role in various stages of cancer progression and is frequently linked to poor prognosis in different human cancers [163,164]. PGE_2_ is notably elevated in CRC, where it is a key mediator of chronic inflammation [165]. The enzyme directly involved in PGE_2_ production, mPGES-1 is highly expressed under inflammatory conditions and overexpressed in adenocarcinomas [166,167]. Hence, it is being considered a potential therapeutic target [168,169]. Particularly notable is the overexpression of mPGES-1 in CRC, often triggered by proinflammatory cytokines like TNF-α [164]. Additionally, mPGES-1 expression can be upregulated by COX-2 overexpression, creating a reciprocal regulatory loop between COX-2 and PGE_2_. This feedback mechanism not only maintains COX-2-driven PGE_2_ production but also allows PGE_2_ to further enhance COX-2 expression in colon cancer cells [100]. As stated above, PGs exert their functions by binding to their specific GPCRs. GPCRs, in general, were shown to participate in the promotion of many cancers, being one of the largest classes of receptors located in the cell surface [170]. The importance of GPCRs, which are often deregulated in cancer development has been underestimated, as the mechanisms behind their effect are far from being elucidated [170,171]. PGE_2_ promotes tumor progression through its association with receptors EP1-4 [163]. EP3 and EP4 are high-affinity receptors, activated by low levels of PGE_2_, while EP1 and EP2 require higher concentrations. This allows PGE_2_ to trigger different cellular responses depending on receptor type, tissue and disease context [172].

Of the four receptors, EP1 has the least affinity for PGE_2_ [173]. It is expressed in many different cancers, such as skin carcinoma [174], CRC [175] and hepatocellular cancer [176]. EP1 receptor activation drives cancer cell migration and invasion through Gαq signaling, which increases intracellular Ca^2+^ and activates PKC, triggering transcription factor pathways like NFAT, NFκB, and MAPK [111]. Its role in cancer is supported by studies in animal models. For example, EP1 inhibition, via selective antagonists or in knockout (KO) mice, is known to reduce early tumor precursors like aberrant crypt foci in azoxymethane- treated mice [177]. EP1 interference lowers polyp formation in APC KO mice (a model of intestinal tumorigenesis) [178] and decreases UVB-induced skin tumors [179]. These findings underscore EP1’s contribution to tumor development across multiple tissues. It also helps tumors, including CRC, to adapt to hypoxia. Under low oxygen, HIF-1 increases COX-2 and PGE_2_ levels, which further enhance HIF-1 activity [152]. EP1 expression is also upregulated in CRC cells during hypoxia, supporting cancer cell survival in this environment [180]. It can also contribute to immune suppression by inducing FasL expression [181,182] and recruiting immunosuppressive cells like Tregs [181] and MDSCs [183]. While most evidence supports a tumor-promoting role, EP1 may have anti-metastatic effects in some breast cancers [184], possibly due to tissue-specific functions. Targeting EP1 may hold therapeutic promise, but its efficacy in humans remains to be shown.

EP2 activation, leading to proinflammatory cytokine release can promote cancer growth, invasion and angiogenesis [91,185]. Overexpression of the EP2 receptor is found in different cancers, including colon cancer, where it enhances cell growth through the β-catenin signaling pathway [91,186,187].

EP3 is the receptor with the highest affinity to PGE_2_ [188] and acts as a double-edged sword in different cancers, mediating carcinogenesis with conflicting effects. EP3 can promote tumor progression by enhancing migration, angiogenesis, and survival through ERK1/2, p53, EGFR, and TGFβ/Smad pathways, by increasing factors like PAI-1, uPAR, VEGF, and MMP9 [189,190,191,192]. In NSCLC, EP3 inhibition reduces viability and migration, promotes apoptosis, and suppresses TGF-β/Smad signaling, highlighting therapeutic potential [193]. Similarly, EP3 antagonism lowers proliferation and migration in breast cancer cells, possibly via decreased Gi-protein signaling and increased cAMP, though further research is needed [190]. In the same way, EP3 expression in glandular epithelial cells is linked to poor prognosis in endometrial cancer, where its inhibition shows anti-cancer effects, making it a promising diagnostic and therapeutic target [194]. In contrast, EP3 is often downregulated in advanced mammary carcinoma [195], CRC [196] and skin cancers [174], with minimal impact on tumor development in some models. In COX-2-driven breast cancers, EP3 is reduced while EP1, EP2 and EP4 are increased, suggesting a protective role of EP3 [197]. Moreover, EP3 does not influence skin tumor formation [198] and its deletion has no impact on CRC progression in APC^min^ mice [199]. This complexity is partly due to multiple EP3 isoforms produced by alternative splicing, which differ in signaling and gene regulation. EP3-Ia, EP3-II, and EP3-III isoforms vary in their ability to induce ERK1/2 phosphorylation and transcriptional activity, influencing their roles in cancer [113]. It has been shown that EP3 might play also a role in angiogenesis. Mice lacking the EP3 receptor (EP3^−/−^) showed significantly reduced tumor growth and tumor-associated angiogenesis compared to wild-type mice after implantation of sarcoma cells. In these mice, a reduction in the expression of VEGF, an essential angiogenesis molecule, was observed [200]. Overall, EP3’s complex and context-dependent roles in cancer make it a challenging target, requiring further studies to clarify its effects across cancers. In general, EP3 might have minimal or even adverse effects on CRC tumorigenesis, as its expression tends to decline in the later cancer stages [91,196].

The EP4 receptor has emerged as a promising therapeutic target in cancer treatment, particularly in CRC. PGE_2_, by activating EP4 receptors in dendritic cells, increases the production of pro-inflammatory cytokines such as IL-17, MCP-1, IL-6, IL-23, and IL-8, which exacerbate inflammation and contribute to CRC development [201]. Additionally, in human colon cancer HCA-7 cells, EP4 receptor activation induces COX-2 expression through signaling mechanisms involving Gαi and PI3K activation. This process is likely involved in the initial stages of tumorigenesis in CRC [202]. Moreover, expression levels of EP4 mRNA have been assessed in both normal and malignant colon cells in mice, supporting the implication of EP4 in colorectal carcinogenesis [203]. Additionally, PGE_2_ production and COX-2 expression are markedly increased in cancer-associated fibroblasts within CRC tissue compared to normal fibroblasts. This is contributing to a tumor-stimulating microenvironment and creating an immunosuppressive setting that hampers anti-tumor immune responses [204,205].

Taken together, PGE_2_ and its receptors, particularly EP1 and EP4, are implicated in driving CRC progression, suggesting that antagonists targeting these receptors may represent a promising therapeutic strategy for CRC treatment. From the wide variety of PGs produced by COX-2 expression upon inflammation, only the contribution of PGE_2_ to tumor development has been intensively researched. Whereas the role of the other PGs is still largely unexplored.

#### 3.5.2. The Uncertain Role of PGF_2α_ and the FP Receptor: Emerging Evidence in CRC

Like PGE_2_, PGF_2α_ exhibits also pathological roles in vivo [206]. AKR1C3, the major PGF_2α_ producing enzyme, has recently been recognized as a key driver of accelerated proliferation and metastasis in carcinomas. Its overexpression acts as an oncogenic factor, promoting tumor cell growth, invasion and metastasis. It is associated with poor prognosis and overall survival in carcinoma patients. However, it has to be underscored that AKR1C3 has also other enzymatic functions in steroid hormone metabolism by regulating androgen and estrogen balance, which is crucial in hormone-sensitive tissues. Furthermore, it is involved in the detoxification of reactive aldehydes and ketones, derived from lipid peroxidation and environmental toxins, ultimately protecting cells from oxidative stress and electrophilic damage [207,208,209]. Inhibiting AKR1C3 has shown strong potential in halting tumor progression and overcoming treatment resistance. This has led to a surge of global interest in AKR1C3 as a therapeutic target, with numerous studies underway to develop AKR1C3 inhibitors [209]. While AKR1C3 is typically upregulated in various cancers, it was described to be downregulated in CRC compared to normal tissues. Li Yafei et al. found that the transcription factor ARID3A suppressed AKR1C3 expression in CRC cells. This was linked to reduced sensitivity to 5-fluorouracil (5-FU), suggesting that the ARID3A-to-AKR1C3 ratio could be a useful prognostic marker for CRC patients undergoing chemotherapy [210]. Moreover, PGF_2α_ plays an important role in the regulation of MMP production, particularly MMP2 [211,212]. The MMP family is a group of enzymes involved in the degradation of the ECM. Specifically, MMP2, being able to degrade type IV collagen, which is a major structural element of the basement membrane (BM), has a key role in cancer invasion and metastasis [213,214]. Taking the crucial role of the BM/ECM into consideration, this would suggest that PGF_2α_ may possess some pro-metastatic abilities [215]. A few studies have identified high levels of PGE_2_, but also of PGF_2α_ in CRC. In a study involving patients with familial adenomatous polyposis (FAP) it was confirmed that PGF_2α_ is produced at higher levels (around 30-fold increase) in patients with FAP than PGE_2_ when analyzing intestinal tissue [216,217]. Moreover, expression analysis revealed that genes induced by PGE_2_ overlapped with those induced by COX-2 activation; but were activated at lower levels, pointing to a potential involvement of alternative COX-2 derived PGs like PGF_2α_ [100]. Intriguingly, elevated PGF_2α_ levels are a distinctive trait in patients with Crohn’s disease [218], which show a higher risk to develop CRC. PGF_2α_ can trigger a concentration-dependent increase in chloride secretion through a cAMP-mediated response in the human colonic epithelium. The amount of cAMP in the crypts of the human colon was also elevated after treatment with PGF_2α_. Taken together, this leads to an inflammatory response involving elevated levels of fluid secretion into the colonic lumen that has implications in the development of inflammatory associated diseases like IBD [124]. Moreover, PGF_2α_, secreted by colorectal adenoma- and carcinoma-derived cell lines, was able to induce cell motility in vitro at the same level of efficacy as PGE_2_. In addition, it was also shown that this particular PG increases the invasiveness of carcinoma cells. Interestingly, PGF_2α_ may not only influence cell motility, but also induce the production of matrix remodeling enzymes and interfere with the cell adhesion complexes [123].

FP has also been implicated in the promotion of tumor development. FP expression has been associated with progression and proliferation of prostate cancer [219] and is considered a potential biomarker for prostate cancer to predict cancer progression after stage II [220]. The FP receptor is also expressed in the epithelium of normal colon, colorectal adenomas and adenocarcinomas [123]. Autocrine PGF_2α_ secretion was able to enhance the expression of mPGES-1 and COX-2 in CRC cells through the FP receptor and EGR1, further promoting PGE_2_ levels. This would indicate a positive feedback loop between COX-2/mPGES1/PGE_2_ and PGF_2α_ [221]. Overexpression of FP was described in tumor endothelial cells in renal cell carcinoma, suggesting a possible link to tumor promoting angiogenesis [222]. As stimulation of the FP receptor by PGF_2α_ triggers the initiation of the MAPK pathway, leading to the activation of ERK1/2 and subsequent activation of different pro-inflammatory transcription factors like NF-κB, it is tempting to speculate that FP activation might contribute to tumor growth, development, cell proliferation and metastasis formation in general [223,224]. It is also important to take into consideration that, while PGF_2α_ mainly signals through the FP receptor, it may exhibit cross-reactivity with EP receptors under certain conditions. For example, PGF_2α_ can have affinity for the EP1 and EP3 receptors, suggesting that it could contribute to signaling pathways typically associated with PGE_2_ [225]. The affinity of PGF_2α_ for the EP receptor is around 100–300-fold less than the PGE_2_ [173]. On the other hand, there might also be an impact of PGE_2_ via FP signaling, as it can bind to the FP receptor with an approximate 10–30-fold less affinity than PGF_2α_ [173].

In summary, PGF_2α_ function in CRC or in tumorigenesis in general is underexplored. There is some evidence for a possible role of PGF_2α_ and the FP receptor in CRC development, but overall, this particular area of research remains unclear with few evidence regarding the mechanism of action and the specific contribution of these molecules to CRC.

#### 3.5.3. PGD_2_ and Its Dual Role in Tumorigenesis

The role of PGD_2_ remains a subject of debate [226]. There are reports supporting its tumor-suppressive properties. For instance, the deletion of H-PGDS has been associated with a marked increase in intestinal polyposis, alongside elevated levels of pro-inflammatory cytokines such as TNF-α. These data suggest that PGD_2_ may play a protective role in regulating inflammation and tumor development. Moreover, PGD_2_ has shown anti-inflammatory effects through 15D-PGJ2 (produced by non-enzymatic dehydration of PGD_2_ [227]), which binds to the DP receptor [162]. PGD_2_ has also been demonstrated to inhibit neovascularization, a critical process for tumor growth and progression [87]. These findings suggest that elevated levels of PGD_2_ may suppress tumor growth and are associated with better survival outcomes, indicating PGD_2_’s potential role as a protective factor against cancer [87]. In contrast, the inhibition of L-PGDS has been associated with cancer development in various organs and has been shown to promote the self-renewal of gastric cancer cells, highlighting the distinct roles of PGD_2_ pathways in cancer progression [91]. Moreover, there is evidence that DP2 receptor expression is significantly increased in tumor tissues of CRC patients compared to normal tissues, while DP1 expression shows the opposite trend. Elevated DP2 expression has been associated with poorer overall survival rates in CRC patients. Those with higher DP2 levels often present with more aggressive tumors, characterized by distant metastasis and advanced stages of the disease, underscoring the pro-tumorigenic role of this receptor [226]. On the other hand, activation of DP1 receptor leads to the activation of protein kinase A, which appears to primarily have anti-inflammatory effects [227]. Especially, TNFα is linked to increased DP2 expression, suggesting its role in shaping the tumor immune microenvironment and contributing to cancer progression. Elevated DP2 levels were also associated with increased metastasis, immune cell migration, and enhanced tumor cell survival and proliferation. These effects may be driven by augmented VEGF, a factor known for promoting survival, migration and invasion in CRC [226,228].

#### 3.5.4. Specialized Prostaglandins: PGI_2_ and TXA_2_ in CRC Development

TXA_2_ has also been associated with inflammation and cancer progression [137,229,230]. Both, TXA_2_ synthesis and expression of its associated receptor are increased in multiple cardiovascular and inflammatory diseases including cancer [137,230]. Thromboxane synthase (TXS), the enzyme transforming PGH_2_ to TXA_2_, is significantly increased in CRC compared to healthy tissue [100]. High levels of TXA_2_ are associated with enhanced proliferation of various cancer cells including CRC, through the activation of the cAMP pathway [91]. Additionally, the progression of CRC correlates with high circulating levels of TXA_2_. This has been demonstrated, for example, in colon tumor-bearing mice displaying up to a 17-fold increase in blood TXA_2_ levels compared to tumor-free controls. Platelets, which are markedly elevated in FAP patients with CRC, are a major source of TXA_2_ in the bloodstream. Importantly, CRC cells can activate platelets to stimulate TXA_2_ production, highlighting the functional role of platelet-derived TXA_2_ in colon tumorigenesis [231,232]. Based on all these results, inhibition of TXA_2_ synthesis or antagonism of the TP receptor might be fundamental in the treatment of many diseases [233]. However, the precise role of TXA_2_ in inflammation remains uncertain [162].

The precise role of PGI_2_ also remains poorly defined, and studies about its involvement in cancer are still at an early stage [91,100]. PGI_2_, produced by PGI synthase (PGIS), exerts its effects via the IP receptor. Some studies indicate that PGI_2_ may promote acute inflammation and worsen chronic inflammation by activating the ERK1/2-MAPK and NF-κB pathways [87,162]. However, lower levels of IP and PGIS expression in CRC were also reported, suggesting that PGI_2_ might have a protective role [87,91,100]. Additional studies propose that PGI_2_ could have anti-metastatic effects due to its ability to inhibit cell proliferation, prevent platelet aggregation induced by tumor cells, and to suppress interactions between cancer cells and endothelial cells [234,235,236].

#### 3.5.5. The Role of 15-PGDH in the Degradation of PGs

The enzyme 15-PGDH, which in humans is encoded by the HPGD gene located on chromosome 4, plays a key role in breaking down PGs and is highly expressed in normal tissues but is often absent in several types of human cancers, including CRC [237]. A two-step mechanism for terminating PG signaling is proposed [116]. Since the presence of 15-PGDH alone in cells was insufficient to oxidize PGE_2_, this model suggests that PGs are first taken up by PG transporter (PGT) proteins across the plasma membrane, followed by their oxidation in the cytoplasm catalyzed by the enzyme 15-PGDH. Indeed, when both PGT and 15-PGDH were co-expressed, rapid oxidation of exogenous PGE_2_ or PGF_2α_ into 13,14-dihydro-15-keto metabolites occurred, supporting the two-step hypothesis [116]. Lastly, these metabolites are excreted via the kidneys [238]. Deficiency of 15-PGDH has been linked to increased CRC growth in mouse models [162,239]. In the last few years, the effectiveness of 15-PGDH as a potential antitumor agent in CRC has been investigated [240,241,242]. Few studies have shown that β-catenin inhibits 15-PGDH expression during colon tumorigenesis, potentially even preceding the upregulation of COX-2 [243]. As a result, the downregulation of 15-PGDH in the normal appearing colorectal mucosa has been linked to sporadic colorectal neoplasia. Downregulation is associated with a more advanced tumor stage and a more synchronous adenoma formation in sporadic CRC patients [244], suggesting a role of 15-PGDH during the early stages of colorectal tumorigenesis. However, 15-PGDH inhibition was also linked to increased liver metastasis and an upregulation of epithelial–mesenchymal transition genes [245]. Interestingly, 15-PGDH expression can also be restored in specific colon cancer cell lines through the reactivation of TGF-β signaling or the inhibition of EGFR signaling [246,247]. Therefore, a promising approach could be to identify compounds capable of reinducing 15-PGDH expression in early neoplastic cells within the gut. These compounds may offer novel, targeted agents with potential effectiveness in preventing colon neoplasia [242,248]. In general, 15-PGDH seems to be involved in the early stages of CRC tumorigenesis but the molecular targets and signaling pathways that interact directly with 15-PGDH in CRC therapy require further investigation to be fully understood.

## 4. Prostaglandin Pathway Inhibitors: Current State of the Art and Therapeutic Perspectives

**Figure 5:**
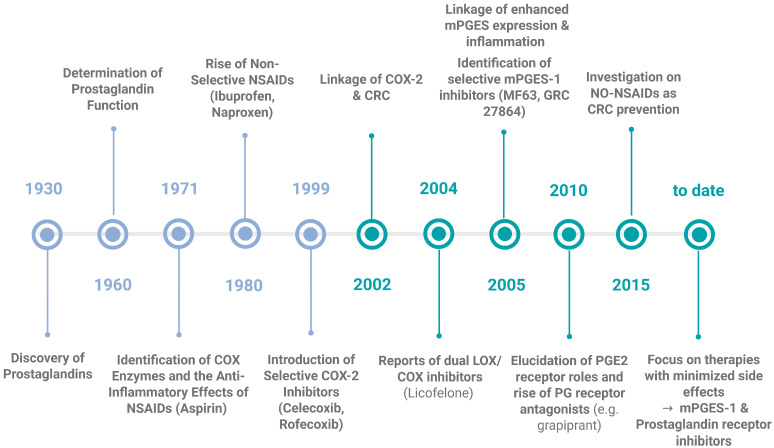
**Milestones in the discovery of prostaglandins, their function and their inhibitors.** This timeline highlights the key scientific breakthroughs, from the initial discovery of the PGs in 1930, till the development of targeted inhibitors and the current state of the art. Current research is focused on the targeted inhibition of mPGES-1 and the understanding and application of prostaglandin receptor antagonists as promising strategies for treating chronic inflammatory conditions and CRC. Created in Biorender. Helmut Dolznig (2025) https://BioRender.com/24z7slp.

One approach to lower the risk of developing CRC is chemoprevention, which is defined as the use of a substance (synthetic or natural) that is able to decrease the possibility of developing cancer, retard the time of cancer initiation, or being able to turn back the tumorigenesis mechanism. So far, only a limited number of chemoprevention agents has been approved by the Food and Drug Administration [249]. One characteristic of a successful candidate is that it should have minimal side effects, because of the long-time frame of substance administration in order to prevent cancer. In general, this type of studies to prevent cancer development are expensive and long in duration, which makes it difficult and challenging to find a perfect candidate [250]. Based on the fact that the majority of CRC cases develop from adenomas, the main aim of chemoprevention is to prevent adenoma formation. Since the process of adenoma formation is likely linked with aberrant PG production [251,252] makes the management of PG levels with chemoprevention so attractive. As stated before, the initial discovery of prostaglandins was made in 1930 [89] and in 1960, their physiological functions were comprehensively characterized, confirming their crucial role in various biological processes. A significant breakthrough occurred in 1971 with the identification of cyclooxygenase (COX) enzymes and the subsequent elucidation of their role in the mechanism of action of nonsteroidal anti-inflammatory drugs (NSAIDs) [253]. As an increase in the levels of COX-2 mRNA and protein are found in most CRC compared to the normal surrounding mucosa and the development of this type of cancer is linked to an overexpression of this enzyme [254], there has been extensive research about inhibiting COX-2 function by NSAIDs or COX-2 inhibitors (COXIBs) to control CRC development during the last few decades (Figure 5). COX-2 inhibitors work by directly obstructing PG synthesis. Synthetic inhibitors have been available for years and are known to effectively suppress COX-2 activity during inflammation. The first generation of NSAIDs included nonselective COX inhibitors such as aspirin, which affected both COX-1 and COX-2 [68]. Clinical studies have showcased that prolonged NSAID use diminishes the relative risk of CRC by 40–50% [239]. Nevertheless, prolonged use of these inhibitors is linked to adverse effects such as nausea, dyspepsia, abdominal pain, as well as gastrointestinal issues like gastritis, peptic ulcers, gastrointestinal bleeding and perforation of gastroduodenal ulcers [255,256,257]. These problems are likely due to the unintended inhibition of COX-1, which is constitutively expressed [68,151]. Consequently, researchers developed selective COXIBs, such as celecoxib (co-developed by G. D. Searle and Pfizer in 1999), which is 300 times more active against COX-2 than it is against COX-1 [151], to minimize these side effects. Although studies have demonstrated that selective COX-2 inhibition can help prevent tumor development or recurrence [258], they have also identified cardiovascular risks associated with these drugs [100]. These side-effects were attributed to (a) the decrease in the PGI_2_, as it participates in the control of platelet aggregation and blood flow, and (b) the increased levels of leukotrienes, both direct consequences of inhibiting the COX-2 pathway [259,260,261,262]. Celecoxib, which was initially approved by the FDA for treating FAP, is no longer recommended for this use [162]. There are still some studies ongoing about the use of celecoxib as an adjuvant to conventional chemotherapy for the treatment of CRC, obtaining mixed results; either being successful or lacking benefits [263,264,265]. Even though in 2002, the link between COX-2 and CRC was proposed, highlighting the enzyme’s importance in cancer biology [266] and many studies have demonstrated decrease in CRC development by inhibiting the COX enzymes (both or specifically COX-2) [267], the risk of their numerous side effects is still too high. Thus, COXIBs have not widely been accepted as the best candidates for CRC chemoprevention, however there is an exception when it comes to high-risk individuals such as patients with FAP or any CRC predisposition syndromes [250].

An alternative possibility would be to target the downstream signaling pathway by, for example, blocking the specific PGs synthases. Some studies performed in mice have found that deletion of the mPGES-1 suppresses or considerably reduces intestinal tumorigenesis [268,269]. A recent report highlighted the therapeutic potential of selective mPGES-1 inhibitors. They demonstrated that the mPGES-1 inhibitor, CIII, effectively reduced PGE_2_ production while increasing levels of PGF_2α_, affecting the lipidomic and proteomic profiles in A549 lung cancer cells, and showing a decrease in cell proliferation and enhancing the effect of other drugs, such as cisplatin, combined with the inhibitor [270]. Some specific inhibitors have been developed since the discovery of mPGES-1 and tested in preclinical settings. However, to the best of our knowledge, so far there is not a single inhibitor approved for clinical trials, mainly because of the interspecies difference in the structure of the enzyme between humans and rodents [271,272].

Combinatorial targeting of different PG producing enzymes in CRC using low doses of inhibitors against each one to avoid the adverse effects or using them as adjuvants to other treatments seems to be a promising approach. Moreover, simultaneously targeting the 5-LOX pathway involved in the formation of inflammatory leukotrienes is regarded as an encouraging option [165,273,274]. In support of this, it has been demonstrated that using a dual combinatory inhibition with celecoxib and AA861, a specific 5-LOX inhibitor, displayed a better response than the use of each inhibitor individually in a CRC xenograft model, reducing side effects because of the use of lower doses [254]. Also, Licofelone, another dual COX/5-LOX inhibitor, induces apoptosis in HCA-7 colon cancer cells [275].

Another strategy could be to block PG receptors by using antagonists. In recent years, there was considerable effort invested to characterize and elucidate the role of each receptor and its mechanism of action by using specific agonists and antagonists [276]. The exploration of PGE_2_ receptor antagonists began in 2010, offering further advancements in therapeutic potential for inflammatory diseases. Recent studies on the EP1 antagonist (ONO-8711) and the EP4 antagonist (ONO-AE2-227; Grapiprant) indicate that EP4 receptor antagonists show greater potential in treating pain, inflammation and solid tumors including CRC [165,277]. The EP4-selective antagonists ONO-AE2-227 and ONO-AE3-208 are undergoing preclinical evaluation and have demonstrated promising effects inhibiting tumor growth in animal models [278,279]; nevertheless, their clinical testing and application remained limited. However, systemic administration of these inhibitors inherently includes the problem of cell type specificity and demand for evaluating safety profiles due to side effects [280].

## 5. Conclusions and Future Directions

PGs are known to play an important role in the development and progression of CRC, primarily by their impact on the inflammatory response and their immune modulation capacity. As stated in this review, among all the PGs the one that has been most extensively investigated for its contribution to CRC progression, is PGE_2_. However, it becomes evident that other PGs substantially influence tumor cells and their microenvironment. More research is needed in this direction, for instance, focusing on PGF_2α_ as a target, being present at high quantities in CRC. On the other hand, there is urgent need to investigate the synthesis patterns and functions of PGs and expression patterns of PG receptors in individual cell types with a special focus on cells of the TME. Further functional research is needed to clarify the role of PGs in the complex interaction of cell types within the TME and the impact on cancer cells.

The principal success of NSAIDs and COXIBs in reducing CRC risk is encouraging and strongly supports the idea of inhibiting PGs action as a strong chemoprevention strategy. However, the clinics is still struggling with the severe side effects. Additionally, in populations regularly screened for CRC chemoprevention studies must show a significantly greater protective effect to demonstrate added benefits beyond screening. In order to overcome this problem, the challenge remains to identify the most selective and effective (combination) therapies, as well as a deeper understanding about the role of the different PGs and their receptors in the context of inflammation and cancer. For public health the ideal chemopreventive agent would be highly effective, safe, affordable, accessible, and easy to use. Although finding such an agent is challenging, the potential to reduce CRC risk and its related health impacts makes pursuing chemoprevention a worthwhile goal and interfering with PG signaling remains a hot candidate in this task.

## Figures and Tables

**Table 1:** Comprehensive list of genes, proteins, enzymes and receptors involved in the prostaglandin pathway: synthesis, metabolism, and signaling.

Gene Name	Symbol	Protein Name	Abbreviation	Product/Ligand
*Phospholipase A2* *Group IVA*	PLA2G4A	Cytosolic phospholipase A2 alpha	cPLA2	Releases arachidonic acid (AA)
*Prostaglandin-endoperoxide synthase 1*	PTGS1	Cyclooxygenase-1	COX-1	Converts AA to PGG_2_/PGH_2_
*Prostaglandin-endoperoxide synthase 2*	PTGS2	Cyclooxygenase-2	COX-2	Converts AA to PGG_2_/PGH_2_
*Prostaglandin D2 synthase*	PTGDS	Prostaglandin D2 synthase	PGDS	Converts PGH_2_ to PGD_2_
*Hematopoietic PGD synthase*	HPGDS	Hematopoietic prostaglandin D synthase	H-PGDS	Converts PGH_2_ to PGD_2_
*Prostaglandin E* *synthase (microsomal)*	PTGES	Microsomal prostaglandin E synthase-1	mPGES-1	PGE_2_ (from PGH_2_)
*Prostaglandin E* *synthase 2*	PTGES2	Prostaglandin E synthase 2	mPGES-2	PGE_2_ (from PGH_2_)
*Prostaglandin E* *synthase 3*	PTGES3	Prostaglandin E synthase 3	cPGES	PGE_2_ (from PGH_2_)
*Prostaglandin F synthase*	AKR1C3	Aldo-keto reductase family 1 member C3	PGFS/AKR1C3	PGH_2_→PGF_2α_; PGD_2_→11β-PGF_2α_
*Aldo-Keto Reductase Family 1 Member B1*	AKR1B1	Aldose reductase/Prostaglandin F synthase	AKR1B1, ALDR1, ALR2	PGH_2_ → PGF_2α_
*Prostaglandin I2 synthase*	PTGIS	Prostacyclin synthase	PGIS	Converts PGH_2_ to PGI_2_ (prostacyclin)
*Thromboxane A* *synthase 1*	TBXAS1	Thromboxane-A synthase	TXAS	Converts PGH_2_ to TXA_2_
*15-Hydroxyprosta-glandin dehydrogenase*	HPGD	15-Hydroxyprostaglandin dehydrogenase	15-PGDH	Inactivates PGs by converting PGE_2_, PGD_2_, and PGF_2α_ into their inactive 13,14-dihydro-15-keto forms.
*Prostaglandin D2 receptor*	PTGDR	Prostaglandin D2 receptor	DP1	Binds PGD_2_
*Prostaglandin D2 receptor 2*	PTGDR2	Prostaglandin D2 receptor 2	DP2/CRTH2	Binds PGD_2_
*Prostaglandin E* *receptor 1*	PTGER1	Prostaglandin E2 receptor 1	EP1	Binds PGE_2_
*Prostaglandin E* *receptor 2*	PTGER2	Prostaglandin E2 receptor 2	EP2	Binds PGE_2_
*Prostaglandin E* *receptor 3*	PTGER3	Prostaglandin E2 receptor 3	EP3	Binds PGE_2_
*Prostaglandin E* *receptor 4*	PTGER4	Prostaglandin E2 receptor 4	EP4	Binds PGE_2_
*Prostaglandin F receptor*	PTGFR	Prostaglandin F2α receptor	FP	Binds PGF_2_α
*Prostaglandin I2 receptor*	PTGIR	Prostacyclin receptor	IP	Binds PGI_2_
*Thromboxan receptor*	TBXA2R	Thromboxan receptor	TP	Binds TxA_2_

## Data Availability

No new data were created or analyzed in this study. Data sharing is not applicable to this article.
